# Comprehensive Genome Analysis on the Novel Species *Sphingomonas panacis* DCY99^T^ Reveals Insights into Iron Tolerance of Ginseng

**DOI:** 10.3390/ijms21062019

**Published:** 2020-03-16

**Authors:** Yeon-Ju Kim, Joon Young Park, Sri Renukadevi Balusamy, Yue Huo, Linh Khanh Nong, Hoa Thi Le, Deok Chun Yang, Donghyuk Kim

**Affiliations:** 1 College of Life Science, Kyung Hee University, Yongin 16710, Korea; huoyue0214@khu.ac.kr (Y.H.); dcyang@khu.ac.kr (D.C.Y.); 2 School of Energy and Chemical Engineering, Ulsan National Institute of Science and Technology (UNIST), Ulsan 44919, Korea; jypark57@unist.ac.kr (J.Y.P.); lizkhanhlinh@unist.ac.kr (L.K.N.); hoale92@unist.ac.kr (H.T.L.); 3 Department of Food Science and Biotechnology, Sejong University, Seoul 05006, Korea; srirenukadevibalusamy@gmail.com; 4 School of Biological Sciences, Ulsan National Institute of Science and Technology (UNIST), Ulsan 44919, Korea; 5 Korean Genomics Industrialization and Commercialization Center, Ulsan National Institute of Science and Technology (UNIST), Ulsan 44919, Korea

**Keywords:** Plant growth-promoting rhizobacteria, Sphingomonas, Panax ginseng, Iron stress, Biotic stress

## Abstract

Plant growth-promoting rhizobacteria play vital roles not only in plant growth, but also in reducing biotic/abiotic stress. *Sphingomonas panacis* DCY99^T^ is isolated from soil and root of *Panax ginseng* with rusty root disease, characterized by raised reddish-brown root and this is seriously affects ginseng cultivation. To investigate the relationship between 159 sequenced *Sphingomonas* strains, pan-genome analysis was carried out, which suggested genomic diversity of the *Sphingomonas* genus. Comparative analysis of *S. panacis* DCY99^T^ with *Sphingomonas* sp. LK11 revealed plant growth-promoting potential of *S. panacis* DCY99^T^ through indole acetic acid production, phosphate solubilizing, and antifungal abilities. Detailed genomic analysis has shown that *S. panacis* DCY99^T^ contain various heavy metals resistance genes in its genome and the plasmid. Functional analysis with *Sphingomonas paucimobilis* EPA505 predicted that *S. panacis* DCY99^T^ possess genes for degradation of polyaromatic hydrocarbon and phenolic compounds in rusty-ginseng root. Interestingly, when primed ginseng with *S*. *panacis* DCY99^T^ during high concentration of iron exposure, iron stress of ginseng was suppressed. In order to detect *S. panacis* DCY99^T^ in soil, biomarker was designed using *spt* gene. This study brings new insights into the role of *S*. *panacis* DCY99^T^ as a microbial inoculant to protect ginseng plants against rusty root disease.

## 1. Introduction

Heavy metal exposure is a major threat to plant and environmental resources worldwide. The growth of industrialization has increased the accumulation of heavy metals in the environment resulting in adverse effects to the soil and crop productivity [1]. Although iron (Fe) is considered as an essential micronutrient for plant growth, metabolism, and development [2], optimum concentration of soils ranges from 0.2% to 55% (20,000 to 550,000 mg/kg) respectively [3]. Thus high concentrations of iron in soil can be detrimental to the yield and growth of plant [4,5]. *P. ginseng* has been widely used in Asian countries as a traditional medicine for thousands of years. Ginseng is a perennial plant, which grows for more than 6 years in humus-enriched soil at a slightly acidic pH (pH 5.0-6.0). Under this acidic environment, heavy metals are readily accessible to ginseng roots [6]. The healthy ginseng root is well documented as possessing various beneficial pharmacological properties including anti-cancer, anti-emetic, anti-oxidative, and anti-angiogenic effects [7,8,9,10]. The pharmaceutical properties of ginseng are attributed to its increased accumulation of secondary metabolites, including ginsenosides. Not only prolonging ginseng cultivation increase ginsenoside accumulation but also cause ginseng rusty root disease [11]. 

Although pathological mechanism of rusty root disease has not been clearly elucidated, some studies suggest that rusty root corresponds to the occurrence of physiological stress. Whereas some studies have linked the amassment of phenolic compounds on the ginseng root surface as a cause of rusty root [11,12,13], another study demonstrated that deposition of iron compounds may be the cause of the disease [5]. However, few studies have examined the soil characteristics linked to the rusty root disease, which is one of the key factors known to affect plant growth. Numerous studies indicates a possible link between rusty root and ginseng soil properties [14], where it was demonstrated that ferrous iron may be one of the foremost factors that cause rusty root disease. Another study showed that active reducing organic substances released under high moisture conditions can activate iron oxides leading to the accumulation of divalent iron. This evidence indicates that the deposition of Fe (II) iron in the ginseng root epidermis might induce the rusty root condition [14,15]. A number of studies also revealed that ginseng root quality is affected by pathogenic bacterial and fungal infection, resulting in reddish-brown to orange-brown discolored regions of the plant root [16]. Therefore, a better understanding of rhizosphere microbial properties in rusty root conditions can contribute to an increased ginseng yield by preventing rusty root conditions under iron stress [17].

In plant cultivation, microbial population is known to play a vital role in increasing or reducing yield [18,19] where specific bacterial populations have been chosen as fertilizers and pesticides to promote plant growth and yield. Microbes that help plant growth are known as plant growth-promoting bacteria. Plant growth-promoting bacteria can stimulate plant tolerance against heavy metal stress by upregulating expression of heavy metal stress-related genes [20]. In addition, the application of plant growth-promoting bacteria in bioremediation has played a significant role in the large scale removal of heavy metal pollutants from soil [21]. Several reports confirmed that certain types of plant growth-promoting bacteria exhibited metal tolerance properties, including *Variovorax, Methylobacterium, Burkholderia, Okibacterium, Rhodococcus, Microbacterium, Sphingomonas, Curtobacterium, Serratia, Pseudomonas, Ralstonia, Staphylococcus, Bacillus, Arthrobacter, Paenibacillus, Chryseobacterium*, and *Leifsonia* [22,23,24,25,26,27,28]. Among these, little is known about their iron-resistance and functional role in ginseng cultivation against iron toxicity. 

In our research, newly isolated rhizobacterium from soil and root of rusty-ginseng is presented, as *S. panacis* DCY99^T^. *Sphingomonas* species are Gram-negative lipopolysaccharide (LPS)-free bacteria that possess glycosphingolipids instead of LPS. These bacteria inhabit not only the soil but diverse environment. In previous studies, various *Sphingomonas* species have been studied to exhibit traits for promoting plant growth. For example, *S.* sp. LK11 and *Sphingomonas paucimobilis* ZJSH1 are known to produce phytohormones such as gibberellins and indole acetic acid and to solubilize inorganic phosphate in soil [29,30]. *S. paucimobilis* EPA505 is known to degrade poly aromatic hydrocarbons [31,32]. Herein, the bacterium *S. panacis* DCY99^T^ will be phenotypically and genotypically characterized. Through genome-scale analysis of *Sphingomonas* strains, we investigate plant growth promoting property of *S. panacis* during abiotic/biotic stress of ginseng plants.

## 2. Results

### 2.1. Reconstruction of the S. panacis DCY99^T^ Genome

**Figure 1:**
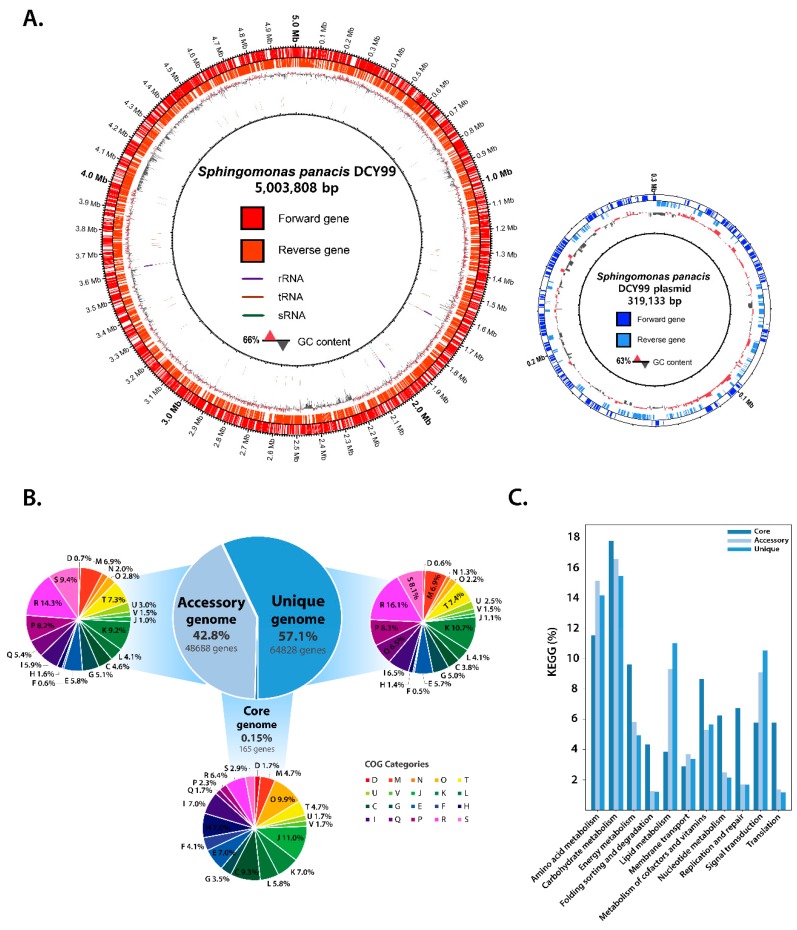
(**A**) Visualization of the circular genome of *S. panacis* DCY99^T^. The genome is shown with a base pair (bp) ruler on the outer ring. The *S. panacis* DCY99^T^ main chromosome is 5,003,808 bp in size; the plasmid is 319,133 bp in size. The chromosome is arranged clockwise. The two outer circles represent *S. panacis* DCY99^T^ CDSs on the forward and reverse strands, respectively. The next circle indicates GC% content and next three circles indicate rRNA, tRNA, and sRNA, respectively. The S. panacis DCY99^T^ plasmid does not contain the three RNAs. (**B**) *Sphingomonas* pan-genome statistics. The *Sphingomonas* pan-genome can be subdivided into three categories: (i) the core-genome (the set of genes shared by all genomes), (ii) the accessory-genome (the set of genes present in some but not all genomes), and (iii) the unique-genome (genes that are unique to a single genome). The function of each gene in a group was classified using COGs. COG categories are as follows. For cellular processes and signaling, D: cell cycle control, cell division, and chromosome partitioning; M: cell wall/membrane/envelope biogenesis, N: cell motility; O: posttranslational modification, chaperones and protein turnover, T: signal transduction mechanisms, U: intracellular trafficking, secretion, and vesicular transport, V: defense mechanisms. For information storage and processing, J: translation, ribosomal structure, and biogenesis, K: transcription, L: replication, recombination, and repair. For metabolism, C: energy production and conversion, G: carbohydrate transport and metabolism, E: amino acid transport and metabolism, F: nucleotide transport and metabolism, H: coenzyme transport and metabolism, I: lipid transport and metabolism, P: inorganic ion transport and metabolism, Q: secondary metabolites biosynthesis, transport, and catabolism, R: general function prediction only, and S: function unknown. (**C**) KEGG pathway distribution of the 159 *Sphingomonas* strains.

The completed genome sequence of *S. panacis* DCY99^T^ was reconstructed using PacBio SMRT sequencing technology as described in the previous report [33]. The genome consists of a single circular chromosome of 5,003,808 bp with a GC content of 65.66% (Figure 1A), which is characteristic of most Sphingomonas strains (60% to 68%). A total of 4,572 genes was predicted, including 62 RNA genes (tRNA:50, rRNA:9, ncRNA:3). There is a single circular plasmid of 319,133 bp with a GC content of 62.71% with a predicted 300 CDSs.

### 2.2. Pan-Genome and Functional Genome Analysis with the 159 Sphingomonas Genomes

In order to better understand physiological properties of *Sphingomonas* for heavy metals resistance and growth-promoting effect on ginseng, pan-genome analysis was performed with the newly sequenced genome of *S. panacis* DCY99^T^ and other public genomic sequences of *Sphingomonas*. A total of 159 *Sphingomonas* genomes including the one for strain DCY99^T^ were analyzed. The resulting pan-genome was further divided by functional characterization of the core and accessory/unique genes (Figure 1B). Unlike the pan-genome of the other bacteria [34], the core-genome, which all of *Sphingomonas* strains share, has only 0.15% of the pan-genome (165 genes), suggesting the genomic diversity of this genus. Functional analysis showed that the core-genome of *Sphingomonas* include genes for translation (J: 11%), post-translational modification/chaperones and protein turnover (O: 9.9%), and energy production/and conversion (C: 9.3%). These results indicate that gene functions for protein synthesis and modification are highly conserved in *Sphingomonas*, suggesting the functional importance of genes in the core-genome. These genes include: *rpl* genes (*rplA, rplI, rplK, rplL, rplM*) and *rpsA, dnaK, clpP, sucA* that encode ribosomal proteins, molecular chaperone, ATP- dependent protease, succinyl-CoA synthetase respectively.

The accessory-genome, which covers 42.8% of the pan-genome (48,688 genes) include genes for transcription (K: 9.2%), inorganic ion transport metabolism (P: 8.2%), signal transduction mechanisms (T: 7.4%), and cell wall/membrane biogenesis (M: 6.9%). Genes encode for transcription and signal transduction mechanisms are commonly found in the core- or accessory-genomes of other bacteria. However, cell wall/membrane biogenesis ranking higher in the accessory-genome is of note for the *Sphingomonas* pan-genome. This is because *Sphingomonas* has been known for its unique type of lipid, sphingolipid [35]. Thus, higher conservation of genes for membrane biogenesis requires further investigation. In addition, two functional groups, general function prediction (R: 14.3%) and function unknown (S: 9.4%), had relatively higher occupancy in the accessory-genome, indicating yet a larger fraction of *Sphingomonas* pan-genome is still elusive for its function. The unique-genome which is not shared by more than two *Sphingomonas* strains, was the largest in the pan-genome. It accounted for 57.1% of the pan-genome (64,828 genes). Similar to the accessory-genome, transcription (K: 10.7%) and inorganic ion transport metabolism (P: 8.3%) were the most relevant functional groups for the unique-genome. In the transcription category of unique-genome, various transcriptional regulators of COG were found, multidrug efflux regulator (AcrR family) [36], regulator related to virulence, metabolism, quorum sensing and motility (LysR family) [37], L-arabinose operons regulator (AraC family) [38], sigma factor for extracytoplasmic function protein family such as metal resistance (RpoE) [39], multiple antibiotic resistance regulator (MarR family) [40,41], and regulator of biofilm formation (CsgD family) [42]. Subsequently, in the inorganic ion transport metabolism category, outer membrane receptor proteins related iron transport, arylsulfatase A and cation diffusion facilitator proteins were found (Supplementary Table S3). Iron transport and cation diffusion facilitator proteins, which show large number of matching genes, suggest the properties of *Sphingomonas* species found in various soil that contain different concentrations of heavy metals. Additionally, arylsulfatase A is involved in the metabolism of organic sulfur and sulfate. Sulfonated polysaccharides is abundant in the marine environment and function as a structural component in marine plants such as *Gelidium* and *Gracilaria* [43,44]. Some of *Sphingomonas* strains are found in the seawater and encode arylsulfatase to utilize these polysaccharides [45]. The diversity of these transcriptional regulators and membrane transport proteins is a possible explanation for the distinct lifestyles of the *Sphingomonas* strains. Consequently, these results suggests that the 159 *Sphingomonas* species can live and adapt to diverse environment niches such as bacterial growth plates [46], seawater [47,48,49], alpine soil [50], spacecraft [51], and arctic-lichen [52]. The differences in these environmental conditions support a rationale as to why the core-genome is so small among these strains. As such, the number of genomes in the core-pan plot increased. While the pan-genome gradually increased, the core-genome remained at a very low fraction (Supplementary Figure S2).

Subsequent analysis of the corresponding core-genome with the KEGG database revealed that enzymatic genes in the core carbon metabolic pathways such as citric acid cycle (TCA cycle) and glycolysis/gluconeogenesis are highly conserved for all *Sphingomonas* strains. For the accessory- and unique-genomes, genes encoding two-component signal transduction systems were frequently found, presumably for the ability to adapt and evolve in various or changing environmental stimuli. In bacteria with several dozen two-component systems, such as *Escherichia coli*, it is clear that the majority of the systems arose by gene duplication from one or more ancestral systems and evolved to acquire new environmental input signals and output specificities [53]. Similarly, genes involved in both fatty acid biosynthesis and degradation pathways were found in the accessory- and unique-genomes. These genes are important for *Sphingomonas* species to produce sphingolipids as a defining characteristic of this particular genus. Indeed, different *Sphingomonas* species produce different lipid compositions including major and minor lipids, which come from different environments and the use of different carbon sources [54]. Therefore, it might be concluded that *Sphingomonas* species use diverse enzymes in lipid metabolism based on the specific niche environment of each strain (Figure 1C). To visualize the evolutionary relationship between the strains of *Sphingomonas*, the pan-phylogenetic tree is constructed (Supplementary Figure S3).

### 2.3. Plant Growth-Promoting Potential of S. panacis DCY99^T^

From the genomic sequence of *S.* *panacis* DCY99^T^ isolated from the roots of *P. ginseng* Meyer was analyzed about enhancing plant growth, production of indole acetic acid, solubilization of phosphate, and antifungal activity. Previous studies have shown that only a few *Sphingomonas* strains are known to promote plant growth through production of phytohormones [55]. Indole acetic acid is the most common phytohormone produced by bacteria and plants. It is one of the auxin class derived from indole which induces cell elongation, cell division and also participates in various gene regulations [56]. 

**Figure 2:**
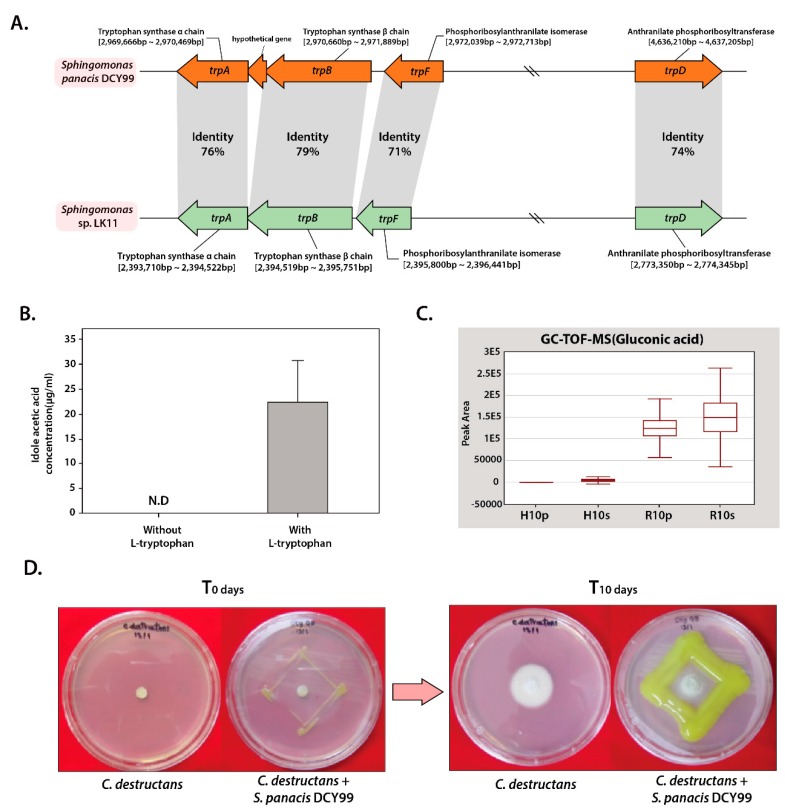
(**A**) Identity of *trp* operon between *S.* sp. LK11 and *S. panacis* DCY99^T^. (**B**) Comparison of indole acetic acid production by the strain DCY99^T^ without L-tryptophan and with L-tryptophan, 22.4 ± 8.37 µg/mL of indole acetic acid is produced from L-tryptophan. (**C**) Results of GC-TOF-MS for D-gluconic acid from epithelium and roots of healthy and rusty 10-year-old *P. ginseng* (Hp, Healthy ginseng epithelium; Hs, Healthy ginseng root; Rp, Rusty-ginseng epithelium; Rs, Rusty-ginseng root). (**D**) In vitro antagonistic test against *C. destructans*. *C. destructans cultured* without and with *S. panacis* DCY99^T^.

*S. panacis* DCY99^T^ was expected that it would show the ability to produce indole acetic acid because several bacteria that colonize the rhizosphere and plant roots can synthesize indole acetic acid from L-tryptophan [57]. However, complete indole acetic acid biosynthesis pathway was not found in strain DCY99^T^ genomic sequence annotation data. Subsequently, comparative analysis using genome data of *S.* sp. LK11, which is known to produce phytohormones such as gibberellins (GAs) and indole acetic acid, was performed [29]. Likewise, complete genes for indole acetic acid biosynthesis were not found in strain LK11. However, the presence of tryptophan biosynthesis gene cluster (*trpA*, *trpB*, and *trpD*), phosphoribosylanthranilate isomerase (*trpF*; locus AWL63_13430), indole pyruvate ferredoxin oxidoreductase (locus AWL63_18305), and indole-3-glycerol phosphate synthase (locus AWL63_21155) which involve in branchpoint of indole acetic acid biosynthesis pathway shows the potential for indole acetic acid production [58]. Previous studies have indicated that the presence of tryptophan-related genes in rhizobacteria is associated with the production of indole acetic acid [59,60]. Thus, the comparative analysis of the tryptophan biosynthesis gene cluster of strain LK11 and DCY99^T^ was carried out. The *trpA*,*B,D,F* genes from *S. panacis* DCY99^T^ were found to have over 70% sequence identity when compared to *S.* sp. LK11 (Figure 2A). Based on the presence of indole acetic acid-related genes with high sequence identity, it is possible that *S. panacis* DCY99^T^ might produce indole acetic acid to promote plant growth, which is similar to strain LK11. Experimental validation of indole acetic acid producing ability of strain DCY99^T^ was performed by growing *S. panacis* DCY99^T^ culture in media with and without additional L-tryptophan. In media with L-tryptophan, *S. panacis* DCY99^T^ produced 22.4 ± 8.37 µg/mL of indole acetic acid whereas in media without L-tryptophan, presence of indole acetic acid was not determined (Figure 2B). As a result, our analysis is confirmed that *S. panacis* DCY99^T^ produces indole acetic acid in a tryptophan-dependent manner.

**Figure 3:**
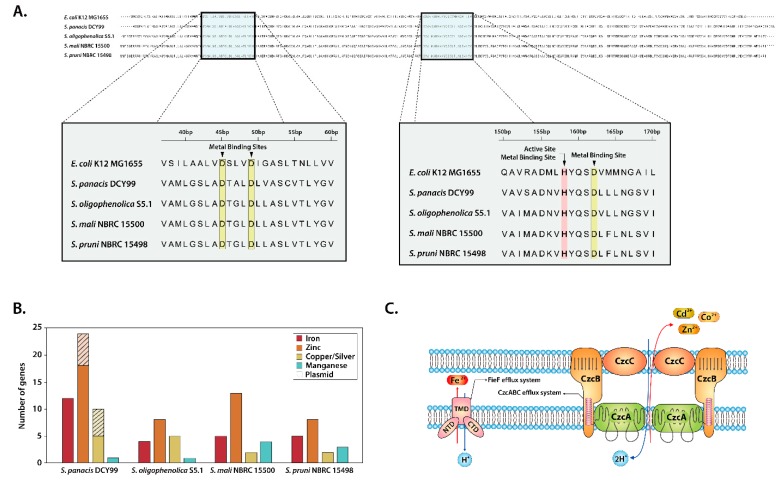
(**A**) Alignment of *fieF* genes from *E. coli* K-12 MG1655 and *S. panacis* DCY99^T^; *Sphingomonas oligophenolica* S5.1; *Sphingomonas mail* NBRC 15500; *Sphingomonas pruni* NBRC 15498. (**B**) Number of heavy metals related genes in four stains of *Sphingomonas*. (**C**) Proposed model for the *czc* efflux system in *S. panacis* DCY99^T^ as suggested *S.* sp. LK11. The *czc* efflux system consist of cell wall “outer” membrane protein (CzcC); “inner” plasma membrane transport protein (CzcA); membrane fusion protein that extends through both membranes (CzcB) [29].

Phosphorus (P) is one of the major essential macronutrients for biological growth and development of plants. Inorganic phosphate is solubilized by plant growth-promoting rhizobacteria, allowing an easy uptake by plant roots [61]. It has been reported that the major mechanism of inorganic phosphate solubilization is the action of organic acids such as gluconic acid and 2-ketogluconic acid synthesized by soil microorganisms [61,62,63,64]. Detailed genomic analysis of *S. panacis* DCY99^T^ was revealed the presence of genes encoding for a complete gluconic acid synthesis pathway, which consists of pyrroloquinoline quinone-dependent glucose dehydrogenase (locus AWL63_22775) and gluconolactonase (locus AWL63_16415) (Supplementary Figure S4A). Interestingly, GC-TOF-MS result of *P. ginseng* Meyer is indicated that gluconic acid concentration is found much higher in rusty- ginseng epithelium and root where *S. panacis* DCY99^T^ was isolated (Figure 3C). Also, when compared with other organic acids, gluconic acid is present at high concentrations in rusty ginseng. In addition, this strain is found to encode the conserved *pst* (phosphate-specific transport) operon that is shown to be responsible for the uptake inorganic phosphate in *E. coli*, and two-component signal transduction system consisting of *phoB*/*phoR* for phosphate transport [29] (Table 1). Phosphate-solubilizing ability of strain DCY99^T^ was tested on Pikovskaya medium. In *S. panacis* DCY99^T^, clear halo regions around colonies which indicate its ability in solubilizing inorganic phosphate was observed.

In our previous study, *S. panacis* DCY99^T^ was reported to exhibit a great antibacterial effect on a rice pathogenic bacteria *Xoo* PXO99Az [33]. The antifungal activity of *S. panacis* DCY99^T^ against *Cylindrocarpon destructans* that cause rusty symptom and root-rot disease of American and Korean ginseng was evaluated [13]. Interestingly, *S. panacis* DCY99^T^ is showed that effectively inhibited the growth of *C. destructans*, which highlights its potential may use for inducing antifungal activity against pathogenic *C. destructans* in ginseng (Figure 2D).

### 2.4. Heavy Metal Resistance of S. panacis DCY99^T^

Metal ions such as zinc, iron, cobalt and manganese are essential for almost all aspects of microbial metabolism. However, excess amount of heavy metals can be toxic to bacteria. Thus, many bacteria have developed efflux mechanisms or resistance to heavy metals [65,66]. Our comparative analysis of *S. panacis* DCY99^T^ with three closely related strains of *Sphingomonas* was revealed the presence of heavy metal related genes and compared with our previous experiment [67]. Despite being one of essential metal, excess iron may affect growth, morphology and metabolism, thus microbes have four groups of different efflux systems [68]. Gene encodes for cation diffusion facilitator *fieF* (also named as *yiiP*) in *E. coli* which survive at high concentration of iron, was also identified in these four strains of *Sphingomonas*. Evidence suggests that *fieF* might be associated with the iron tolerance and full resistance to iron intoxication (Figure 3C) [69,70,71]. *fieF* genes from four strains of *Sphingomonas* and *E. coli* K-12 MG1655 were aligned. The analysis of alignment showed three aspartate residues and one histidine residue being highly conserved in the active site and metal-binding sites (Figure 3A). The conservation of significant amino acid residues in the fieF transport protein substantiates our previous analysis that the ability of *Sphingomonas* to grow under high iron conditions (~2 mM) (Table 2). Additionally, the czc efflux system was found in strain DCY99^T^ genome and plasmid, consists of *czcA*, *czcB*, *czcC* and *czcD*. The czc operon was reported to confer resistance to cobalt, zinc and cadmium. While there are several czc efflux system models for different bacteria, S. panacis DCY99^T^ was shown the highest identity with the model of *S.* sp. LK11 [29,72,73]. This czc model exists as a dimmer and efflux three type of heavy metals (Figure 3C).

In addition, copper resistance genes were identified in S. panacis DCY99^T^ genome. Multi-copper oxidase encoded in S. panacis DCY99^T^ is an important gene for copper resistance in Gram-negative bacteria such as *E. coli*, thus multi-copper oxidase is considered as a marker gene for copper-resistant bacteria [29,74,75]. Moreover, copper-transporting P-type ATPase was identified in the genome analysis, which especially found in bacteria that are resistant to copper [76,77]. Altogether, the number of genes involved in the uptake and resistance of each heavy metal in four strains of *Sphingomonas* genomes was quantified (Supplementary Table S2). Except genes related to manganese, S. panacis DCY99^T^ showed the largest number of heavy metal related genes *(*Figure 3B). Interestingly, strain DCY99^T^ genome is revealed to encode various genes including iron uptake regulator *fur*, iron transporter feoB, and several types of ferrichrome-iron receptors, ferric siderophore transporters, compared to other three strains. Furthermore, its plasmid encodes several heavy metal resistance genes such as related zinc, copper and silver, which was different from the other three strains of *Sphingomonas*. In particular, complete cus operon (cusC; locus AWL63_23365, cusB; locus AWL63_23370, cusA; locus AWL63_23375, cusF; locus AWL63_23380) was found in strain S. panacis DCY99^T^ plasmid. Copper/silver resistant Gram-negative bacteria have *cus* operon which are expressed under high concentration of copper/silver. In *E. coli*, the *cusCBA* efflux complex confers resistance to toxic copper and silver [78,79,80]. The presence of several heavy metals related genes implies that *S. panacis* DCY99^T^ may survive in the polluted soil with high concentration of iron, which associates to rusty root disease in ginseng.

### 2.5. Improvement of P. ginseng Meyer Growth with S. panacis DCY99^T^ under Biotic/Abiotic Stress

**Figure 4:**
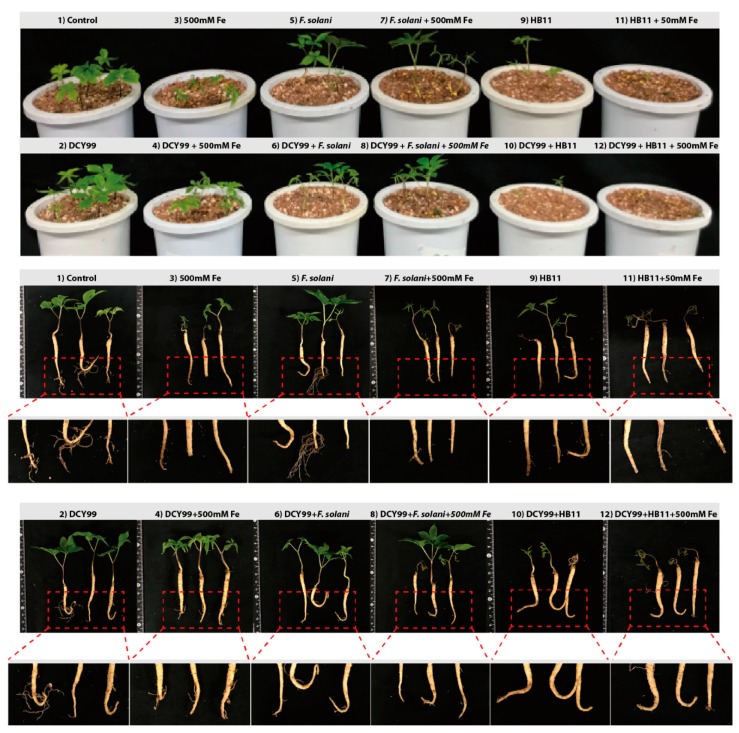
Pot assessment of iron tolerance in *P. ginseng* and resistance against the fungal infection. Morphological appearance of *P. ginseng* in response to iron stress and fungal infection. Pot assay was observed by morphological alterations after 7 days of bacterial inoculation, each pot included five seedlings. (1) Control; (2) *P. ginseng* seedlings inoculated with *S. panacis* DCY99^T^; (3) Control under 500 mM iron stress; (4) *P. ginseng* seedlings inoculated with *S. panacis* DCY99^T^ under 500 mM iron stress; (5) *P. ginseng* seedlings inoculated with *F. solani*; (6) *P. ginseng* seedlings inoculated with *F. solani* and *S. panacis* DCY99^T^; (7) *P. ginseng* seedlings inoculated with *F. solani* under 500 mM iron stress; (8) *P. ginseng* seedlings inoculated with *F. solani* and *S. panacis* DCY99^T^ under 500 mM iron stress; (9) *P. ginseng* seedlings inoculated with *I. mors-panacis* HB11; (10) *P. ginseng* seedlings inoculated with *I. mors-panacis* HB11 and *S. panacis* DCY99^T^; (11) *P. ginseng* seedlings inoculated with *I. mors-panacis* HB11 under 500 mM iron stress; (12) *P. ginseng* seedlings inoculated with *I. mors-panacis* HB11 and *S. panacis* DCY99^T^ under 500 mM iron stress. During iron exposure, the aerial parts and roots of ginseng plants were visibly stressed, however, when the seedlings were primed with *S*. *panacis* DCY99^T^ at the time of iron exposure, iron tolerance was exhibited. But, *S. panacis* DCY99^T^ did not fully confer antifungal effect to seedlings.

Rusty root of ginseng is related to the ecological conditions of the soil and metabolism of the plant caused by excessive iron in the soil, which are mainly composed of organic complex iron species [14]. The compatibility of *S. panacis* DCY99^T^ with *P. ginseng* Meyer under abiotic/biotic stress was tested to determine the effect of strain DCY99^T^ in high Fe (II) concentration conditions and fungal infection. Prior to the abiotic/biotic stress experiment, the pathogenicity of *S. panacis* DCY99^T^ in ginseng was confirmed. *P. ginseng* was dipped in *S. panacis* DCY99^T^ suspensions to inoculate at the root surface of ginseng. Then, ginseng seedlings were cultivated on sterilized artificial soils. The compatibility of strain DCY99^T^ with *P. ginseng* using a pot assay was observed by morphological alterations after 7 days of bacterial inoculation. As a result, *S. panacis* DCY99^T^ showed symbiosis with no significant effect on the growth of *P. ginseng* during the pot assay (Figure 4). Accordingly, the experiment was performed to confirm *S. panacis* DCY99^T^ can affect ginseng growth against iron stress and fungi infection using a pot assay. During the high concentrations of iron exposure (500 mM), the aerial parts of ginseng plants were visibly stressed, and root color changed. *P. ginseng* suffered morphological and physiological changes that were noted in previous studies [5,11]. Interestingly, the morphology of seedlings inoculated with *S. panacis* DCY99^T^ was drastically different. *S. panacis* DCY99^T^ showed significantly suppressed iron stress of ginseng, leading to increased root, shoot and leaf development when compared with seedlings without *S. panacis* DCY99^T^. The compatibility of *Fusarium solani* with *P. ginseng* was assessed by investigating morphological alterations in fungal infection. *F. solani* is known to cause root rot or rusty root disease in ginseng [81]. No disease symptoms were observed in seedling with *F. solani*. However, *S. panacis* DCY99^T^ showed to suppress iron stress of ginseng even if inoculated with *F. solani*. To further confirm the antifungal activity of *S. panacis* DCY99^T^ in ginseng, highly aggressive *Ilyonectria mors-panacis* HB11 which is known to cause aggressive root rot was used in biotic stress [82]. Seedlings with *I. mors-panacis* HB11 were inoculated, after 7 days their root morphologies were slightly altered. In particular, brown discoloration was observed at the tip of the tap root and the foliar growth of plants was significantly affected. Seedling with *I. mors-panacis* HB11 and *S. panacis* DCY99^T^ showed a slightly more stressed to aerial parts of ginseng. Furthermore, growth of ginseng under iron stress with *I. mors-panacis* HB11 was fully inhibited as those seedlings displayed discoloration on their stems and leaves. In addition, their roots were softened with brown discoloration. For seedlings with *I. mors-panacis* HB11 and *S. panacis* DCY99^T^ treatment group were morphologically similar to seedlings with *I. mors-panacis* HB11. However, the morphology of roots inoculated with *S. panacis* DCY99^T^ was not affected by stresses as much when compared with the *I. mors-panacis* HB11 infected seedlings. Consequently, *S. panacis* DCY99^T^ was confirmed to protect seedlings against iron toxicity, thereby preventing developmental inhibition of ginseng under abiotic stressful environment. However, *S. panacis* DCY99^T^ did not fully protect seedlings from fungal infection.

### 2.6. Phenolic Compounds and 3-hydroxybutanoic Acid Degradation by S. panacis DCY99^T^

*S. panacis* DCY99^T^ was found in contaminated soil containing such as benzene, phenolic compounds, and polyaromatic hydrocarbons (PAHs). This strain may have a phenolic compounds-degrading ability when grown on minimal medium containing phenolic compounds. When cultivating ginseng, phenolic compounds such as 4-hydroxybenzoate, vanillin, syringic acid, vanillic acid, coumaric acid, ferulic acid, cinnamic acid, salicylic acid, and benzoic acid were accumulated in the surrounding soil or on the surface of ginseng [83]. The total phenolic content of rusty-ginseng was significantly higher (53%) than that of healthy ginseng roots [84]. The increase of phenolic compounds is consistent with the assumption that phenolics are a part of the cause of rusty-ginseng [84]. Some of *Sphingomonas* strains have been shown to grow on polyaromatic compounds, also strains from different environments have shown the ability to degrade low and high molecular weight polycyclic aromatic hydrocarbons [85,86,87,88,89].

**Figure 5:**
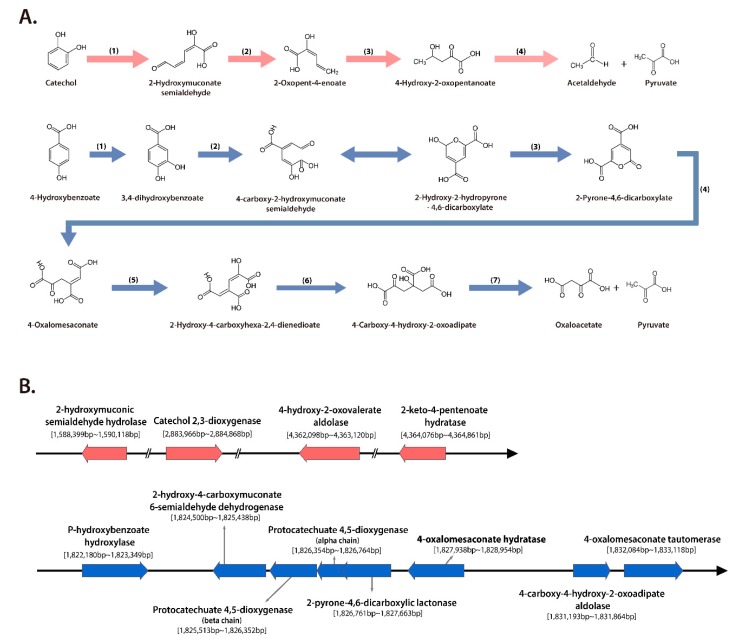
(**A**) Catechol *meta* cleavage and 4-hydroxybenzoate degradation pathways by *Sphingomonas* species. Catechol *meta* cleavage pathway consist of four genes: (1) Catechol 2,3-dioxygenase; (2) 2-hydroxymuconic semialdehyde hydrolase; (3) 2-keto-4-pentenoate hydratase; (4) 4-hydroxy-2-oxovalerate aldolase. 4-hydroxybenzoate degradation pathways contain seven genes: (1) P-hydroxybenzoate hydroxylase; (2) Protocatechuate 4,5-dioxygenase; (3) 4-carboxy-2-hydroxymuconate-6-semialdehyde dehydrogenase; (4) 2-pyrone-4,6-dicarboxylic acid hydrolase; (5) 4-oxalomesaconate tautomerase; (6) 4-oxalomesaconate hydratase; (7) 4-carboxy-4-hydroxy-2-oxoadipate aldolase. (**B**) Position of genes that encode catechol *meta* cleavage and 4-hydroxybenzoate metabolism from *S. panacis* DCY99^T^ genome.

Functional analysis based on protein homologies was performed using *S. panacis* DCY99^T^ with *S. paucimobilis* EPA505. Strain EPA505 was reported to have degraded various polyaromatic hydrocarbons (PAHs) and phenolic compounds [31,32,89]. *Sphingomonas* species use catechol *meta* cleavage pathway that is one of the significant pathways for PAHs degradation (Figure 5A). *S. paucimobilis* EPA505 encodes catechol meta cleavage pathway and 4-hydroxybenzene degradation genes (Table 3) [90]. Genes of degradation pathways of strain EPA505 were used to confirm the presence of protein homologies in the strain DCY99^T^. *S. panacis* DCY99^T^ was confirmed to have complete catechol cleavage pathway genes (locus tag AWL63_07130, AWL63_13025, AWL63_19815, AWL63_19825) in the genome (Figure 5B). Additionally, conserved pathway of degrading 4-hydroxybenzoate was observed. These eight genes are clustered around 1,822,180 bp to 1,833,118 bp on the chromosome (locus tag AWL63_08150, AWL63_08160, AWL63_08165, AWL63_08170, AWL63_08175, AWL63_08180, AWL63_08195, AWL63_08200) and may confer degradation ability from 4-hydroxybenzoate to pyruvate and oxaloacetate. It should be noted that these phenolic compounds degradation genes can be used by *S. panacis* DCY99^T^ to survive on rusty-ginseng surfaces where phenolic compounds are typically present in high concentrations.

Additionally, GC-TOF-MS results of 3-hydroxybutanoic acid was found to be 10 times higher in rusty-ginseng than in healthy ginseng soil (Supplementary Figure S4B). The pathway for degrading 3-hydroxybutanoic acid to two acetyl CoA was found in strain DCY99^T^ genome. This pathway consists of three genes and various Acetyl-CoA acetyltransferase (locus tag AWL63_01810, AWL63_07945, AWL63_07945) (Supplementary Figure S4C). *S. panacis* DCY99^T^ was found in soils that caused ginseng damage due to the phenolic compounds that accumulated in the soil when the medicinal root crops such as ginseng or bellflower were grown for a long time [67]. These results suggest that *S. panacis* DCY99^T^ can survive in the presence of phenolic compounds and will act as a basic indicator for measuring cultivated soil conditions to prevent ginseng root disease.

### 2.7. Design of a S. panacis DCY99^T^ Biomarker

**Figure 6:**
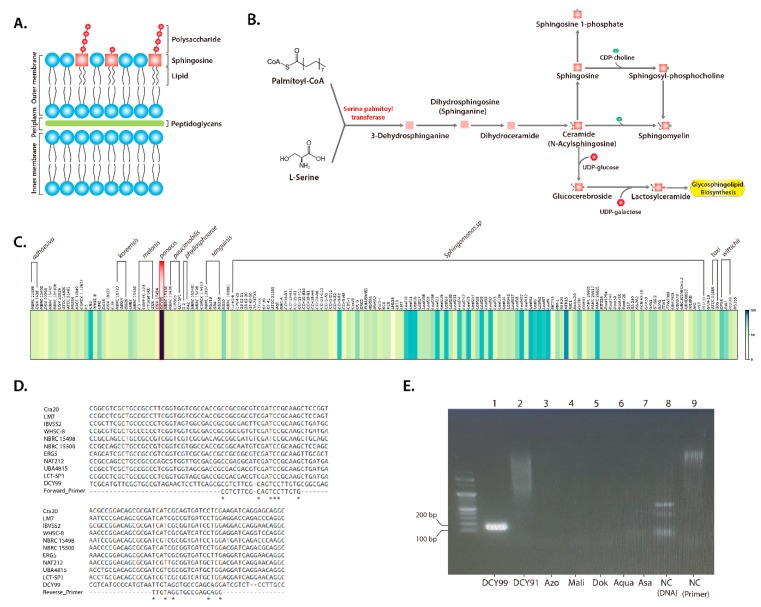
(**A**) LPS-free gram-negative bacterial cell wall structure of *Sphingomonas* species. (**B**) Sphingolipid pathway data from the KEGG database. (**C**) Heat map based on *spt* DNA sequences of the 159 *Sphingomonas* strains for gene of serine palmitoyltransferase from strain DCY99^T^. The sequences of *spt* gene are from the KEGG database. (**D**) Sequence alignment of the *spt* primer for *S. panacis* DCY99^T^ and 10 different *Sphingomonas* strains. (**E**) Quantitative real-time PCR (RT-PCR) analysis of the *spt* primer with 8 *Sphingomonas* strains. Lanes-1 and 2, *S. panacis* DCY99^T^ and *Sphingomonas panaciterrae* DCY91; Lane-3, *Sphingomonas azotifigens*; Lane-4, *S. mali*; Lane-5, *Sphingomonas dokdonensis*; Lane-6, *Sphingomonas aquatilis*; Lane-8, negative control with template DNA, and Lane-9, negative control with primer.

Most microorganisms do not produce sphingolipids [91]; however, *Sphingomonas* species are capable of producing sphingolipids in lieu of LPS. As the name implies, *Sphingomonas* species are gram-negative lipopolysaccharide (LPS)-free bacteria that utilize glycosphingolipids instead of LPS. Glycosphingolipids have remarkable structural similarities with LPS and its hydrophobic characteristics [35] (Figure 6A). This is one of reasons why *Sphingomonas* species are specialized in sphingolipid metabolism [92]. The major sphingolipid biosynthesis pathway, summarized from the KEGG database, is illustrated (Figure 6B). This pathway synthesizes sphingolipid and glycosphingolipid from Palmitoyl-CoA and L-Serine. The heat map is represented the conservation level of *spt* gene (EC:2.3.1.50), the initial step of sphingolipid synthesis is the condensation reaction from cytosolic serine and palmitoyl-CoA to 3-dehydrosphinganine. Result of conservation analysis based on DNA sequences, the *spt* gene was observed with >80% identity (Figure 6C). Additionally, the *spt* gene was verified that it is found in almost *Sphingomonas* strains. Therefore, the *spt* gene was first considered as a key gene for the sphingolipid pathway to design a RT-PCR primer for a *S. panacis* DCY99^T^ biomarker.

A set of *spt* detection RT-PCR primers was designed from sequence alignment with the *spt* sequences of closely related *Sphingomonas* strains and *S. panacis* DCY99^T^ (Table 4), resulting in primer sequences specific only to strain DCY99^T^ (Figure 6D). The result of RT-PCR using the *spt* primer for 7 *Sphingomonas* strains including *S. panacis* DCY99^T^ confirmed the specificity of strain DCY99^T^ *spt* detection primers (Figure 6E). The strain-specific detection marker using the *spt* gene was verified by the 149 bp PCR product that distinguishes *S. panacis* DCY99^T^ from other closely related *Sphingomonas* strains.

## 3. Discussion

The purpose of this study was to determine whether *S. panacis* DCY99^T^ can promote ginseng growth under high concentrations of iron. In general *Sphingomonas* are gram-negative bacteria with more than 103 species [90]. Morphologically, they can be identified as a yellow-pigmented, non-motile, and non-fermentative rod [93]. The outer membrane of all *Sphingomonas* species utilize glycosphingolipids instead of lipopolysaccharides [35]. They are known to confer abiotic stress tolerance in plants [29] and biodegradation of polyaromatic hydrocarbon contaminants [86,87,88,89,94]. Plant growth-promoting rhizobacteria play an essential role in the development and growth of plants [95], in which *Sphingomonas* is often the microbe of interest for their plant growth-promoting activity in *P. ginseng*. Herein, *S. panacis* DCY99^T^ isolated from rusty root ginseng and soil, showed significant growth-promoting effects on *P. ginseng* under high concentrations of iron stress.

The growth-promoting strain DCY99^T^ was phenotypically and phylotypically identified as *Sphingomonas panacis*. Furthermore, strain DCY99^T^ is the most closely related to *S. oligophenolica*, followed by *S. asaccharolytica*, *S. mali*, *S. cynarae*, *S. pruni*, and *S. glacialis* [67]. For a better understanding of rhizobacteria, it is crucial to analyze their genome properties and related genes responsible for adaptation to specific conditions. The genome of strain DCY99^T^ consists of 5.0 Mb chromosomal genome and 315 kb plasmid with 4,810 genes predicted. Of which, 1,725 (36%) genes had their functions classified as hypothetical proteins which have their existence predicted, but there is a lack of experimental characterization [96]. As compared to other bacteria such as *E. coli* and *Salmonella* (<10%), this is a high number of hypothetical proteins. These obstacles make genomic analysis difficult but suggest the potential of discovering novel proteins with function of interest [97,98,99].

To clarify the relationship between *Sphingomonas* strains, the pan-genome analysis using BPGA (Bacterial Pan Genome Analysis) was carried out. BPGA performs pre-processing step to prepare sequence data for clustering. BPGA then runs USEARCH for the fastest clustering using 50% sequence identity cut-off [100]. The USEARCH algorithm is performed in these order: (i) A total of 755,014 genes were assembled from 159 species of *Sphingomonas*; (ii) Out of 755,014 genes, 113,682 genes were clustered in which each cluster is defined by one sequence, known as the centroid or representative sequence; (iii) The USEARCH algorithm used clusters to match one or more sequences from the total 755,014 genes, every sequence in the cluster must have similarity above a given identity threshold (>50%) with the centroid; (iv) Finally, the pan-genome analysis compiled a set of core genes that are clusters containing 159 sequences, accessory genes present in at least two or more sequences, and unique genes only found in a single sequences. The total number of core genes was 165 (0.15%), which was obviously lower as compared to other studies [34,101]. This difference may be due to the higher number of strains analyzed in this study and there was a rich diversity among these strains, including geographical and environmental diversity. Furthermore, the main concern was the quality of genome data of *Sphingomonas* downloaded from NCBI might be the reason for lower number of core genes. This is because if just one of the 187 *Sphingomonas* strains does not have a target gene, the core gene is considered to be an accessory gene. Of the 187 *Sphingomonas* genomes, there were only 15 complete genomes including strain DCY99^T^ while the rest consist of whole-genome shotgun (WGS) sequencing data that contain several contigs. To confirm this problem, the pan-genome analysis using complete genomes of 15 strains was performed. There was a total of 25,745 centroids defined. Of which, 952 (3.69%) core genes, 15,148 (37.46%) accessory genes and 9,645 (59%) unique genes were identified. The number of core genes for 15 complete genomes increased considerably compared to the result of 159 genomes. As the number of analyzed genomes decreases, the number of core genes increases as expected. However, the number of core genes from 15 *Sphingomonas* complete genomes was still significantly less than that of other bacteria (Supplementary Table S3). In addition, the distribution of unique genes was similar to the results that used 159 strains. Therefore, 159 genomic data were used since our purpose in the pan-genome analysis was to focus on the diversity of the *Sphingomonas* genus. In short, these results supported that *Sphingomonas* can constantly obtain foreign genes in order to adapt to various environments [102].

The growth-promoting mechanism of strain DCY99^T^ was determined to be due to a combination of indole acetic acid production, phosphate solubilization and antifungal activity. Indole acetic acid, the physiologically most active phytohormone in plants, acts as an important signaling molecule in the regulation of plant development [103]. Three main biosynthetic routes involving indole-3-pyruvic acid (IPyA), tryptamine (TAM), and indole-3-acetonitrile (IAN) have been studied in plant-associated bacteria [104]. Genes encoding for indole acetic acid biosynthetic pathways from *Pseudomonas savastanoi*, *Enterobacter cloacae*, *Azospirillum brasilense* were analyzed by USEARCH program to identify homologs in *S. panacis* DCY99^T^. However, no indole acetic acid biosynthetic pathways were observed. Therefore, comparative analysis of *S. panacis* DCY99^T^ and *S.* sp. LK11 which is known to be a plant growth-promoting bacteria was performed [29]. This analysis supported that *S. panacis* DCY99^T^ is a strain with great potential in promoting plant growth by producing indole acetic acid from L-tryptophan and solubilizing inorganic phosphate using organic acid such as gluconic acid. Several studies have revealed that ginseng root quality is affected by pathogenic bacteria and fungus [16,105]. Therefore, the ability to compete against pathogenic bacteria and fungus that harm ginseng plants supports a high possibility of plant growth-promoting rhizobacteria traits. Previous study has reported that strain DCY99^T^ shows antibacterial effect on Xoo PXO99Az also known as rice pathogenic bacteria [33]. This study also suggested that *S. panacis* DCY99^T^ has great antifungal ability against *C. destructans* that cause rusty symptom and root-rot disease.

Various studies have reported the heavy metal stress tolerance ability of plant growth-promoting bacteria belonging to different genera [29,106,107]. During exposure to heavy metal stress, these bacteria not only sustain their growth but also exhibit certain plant growth-promoting traits like production of phytohormones, suppression of abiotic/biotic stress [29,106,107]. In the presence of high concentrations of iron, the growth of strain DCY99^T^ was found to be sustained, compared to 3 closely related strains based on their 16S rRNA gene sequences [67]. Comprehensive genome analysis of *S. panacis* DCY99^T^ suggests that this strain has cation diffusion facilitator *fieF* similar to *E. coli* K-12 MG1655 and various heavy metal tolerance genes in genome and plasmid.

The assessment of the abiotic/biotic stress of 2-year-old *P. ginseng* with *S. panacis* DCY99^T^ showed that the iron stress in ginseng with strain DCY99^T^ was significantly suppressed as compared to ginseng growing under sole iron stress treatment. This was verified by the gradual increase in levels of iron concentration before the inoculation of strain DCY99^T^. Seedlings were stressed with different concentrations of iron (0, 250, 500, and 1000 mM) based on previous research [15,108]. The growth of ginseng seedlings was completely disturbed when they were exposed to 1000 mM Fe (Supplementary Figure S5A). Symptoms appeared to be consistent with those seen in the plants suffering from iron toxicity where root and shoot growth was inhibited and the plant biomass decreased [11,109,110]. At 500 mM Fe, ginseng seedlings gradually developed yellowing on the leaves to eventually a complete wilting of the foliage. Biomass reduced after iron stress (500 mM). Fresh weight of root and shoot decreased by 37% and 81% whereas dry weight of root and shoot decreased by 35% and 48% respectively (Supplementary Figure S5B). It was established that at 250 mM Fe, no significant difference was observed when compared to the control [108]. Therefore, 500 mM iron was used as the abiotic stress in this study. When the seedlings were primed with strain DCY99^T^ under high concentration of iron, ginseng was conferred resistant of iron from *S. panacis* DCY99^T^. However, the mechanism of this phenomenon is unclear. One hypothesis is proposed about *S. panacis* DCY99^T^ which survives in iron stress condition, produces gluconic acid - a powerful chelating agent to remove heavy metals [111]. It is established that soil microorganisms produce large amounts of gluconic acid when inorganic phosphates are solubilized [112,113]. Therefore, the gluconic acid produced by strain DCY99^T^ may facilitate the growth of ginseng under high iron stress. In our studies, *S. panacis* DCT99^T^ was reported to have antibacterial and antifungal activity. The antifungal activity of *S. panacis* DCY99^T^ against *I. mors-panacis* HB11 and *F. solani* was validated using *in vitro* antagonistic test. Although strain DCY99^T^ did not completely protect ginseng seedlings from fungal infection in pot assay, it reduced the morphological change of root. Therefore, *S. panacis* DCT99^T^ exhibited a good protection of ginseng from high levels of iron, and a partial protection of ginseng against fungal infection.

*S. panacis* DCY99^T^ can be found from ginseng root to contaminated soil containing benzene, phenolic compounds and polyaromatic hydrocarbons. The analysis of based on protein sequence homologies was proved the presence of PAHs and phenolic compounds degradation pathway in strain *S. panacis* DCY99^T^ as well as strain *S. paucimobilis* EPA505. It was reported that strain EPA505 could grow on various PAHs and partially degrade high molecular weight PAHs [31,32,89]. Moreover, it has well-conserved pathways that degrade PAHs and phenolic compounds. Except for catechol 2,3-dioxygenase (EC 1.13.11.2), which has 30% identity, catechol *meta* cleavage pathway genes showed more than 50% identity. *S. panacis* DCY99^T^ has shown to have higher identity to the 4-hydroxybenzene degradation pathway than the catechol *meta* cleavage pathway compared to *S. paucimobilis* EPA505 (Table 3). These results suggest that *S. panacis* DCY99^T^ also has a high potentiality of surviving even in the presence of these compounds, also, *S. panacis* DCY99^T^ is predicted more likely to grow in 4-hydroxybenzene than PAHs.

In our previous study of *Sphingomonas* species, the phylogenetic analysis through 16S rRNA sequence was conducted [67]. However, *S. panacis* DCY99^T^ shared the highest 16S rRNA gene sequence identity with *S. oligophenolica* JCM 12082T (97.32%), followed by *S. asaccharolytica* KCTC 2825T (96.90%), *S. mali* KCTC 2826T (96.82%), *S.* cynarae JCM 17498T (96.76%), *S. pruni* KCTC 2824T (96.75%), and *S. glacialis* DSM 22294T (96.45%). This high identity can cause the reliability of the biomarker to be lowered. For these reasons, biomarker for strain DCY99^T^ was constructed using *spt* gene that key enzyme of sphingolipid biosynthesis. The *spt* gene is found in all *Sphingomonas* strains and has more variation between species because identity over species is less than 80%. Our RT-PCR result supports that biomarker using *spt* gene can be used to detect strain DCY99^T^ with high reliability. Consequently, *S. panacis* DCY99^T^ will be made an eco-friendly strategy of ginseng cultivation through follow-up studies.

## 4. Materials and Methods

### 4.1. Sphingomonas Strains and Genomic Analysis

A total of 187 *Sphingomonas* genomes were obtained from the NCBI database and used to estimate the genome size/number of DNA coding sequences (CDS) in *S. panacis* DCY99^T^. The trend line *y* = 0.0008x + 737.57 (*R²* = 0.7996) was used to exclude genomes with genes with more than +/− 10% difference (Supplementary Figure S1). The *Sphingomonas* genome was compared with *E. coli* K-12 MG1655 *rpo* genes using USEARCH and we excluded *Sphingomonas* genome data with 0% coverage of the *rpoB* and *rpoD* genes. As a result, only 159 strains were selected for analysis. 

### 4.2. Plant Material and Culture Conditions

Two-year-old *P. ginseng* Meyer was obtained from the Ginseng Resource Bank, Kyung Hee University. To grow ginseng plants, artificial soil was prepared by mixing vermiculite, perlite, and peat moss at a 3:1:1 volume ratio. The mixed soil was autoclaved at 121 °C for 1 h and then air-dried. The sterilization step was repeated twice on different days. Before culturing the roots, tap water was sterilized to prepare the soil mixture at a 25% *v/v* ratio that was used to fill trays or pots for ginseng growth. Each tray or pot was placed in a 60 cm × 100 cm (0.6 m^2^) open cabinet inside a closed controlled chamber. We adjusted the photoperiod to a light:dark (16:8h) cycle using lamps (Philips TLD-RS-FLR32SSEX-D 865K) equal to 9500 lux for each covered area. The temperature was controlled at 25 ± 2 °C, and the moisture level was maintained at 60 ± 5%. Sterilized tap water was sprayed onto the soil surface daily, and watering was conducted once a week using sterilized tap water from a plate beneath the tray/pot.

### 4.3. S. panacis DCY99^T^ Genome Analysis

The *S. panacis* DCY99^T^ genome was deposited as SAMN04417200 in BioSample and as CP014168 for the genome and CP014169 for the plasmid in GenBank. Gene annotation revealed 4601 coding sequences (CDSs) using the RAST (Rapid Annotation using Subsystem Technology) server (Supplementary Table S1). The chromosome (CP014168.1) and plasmid (CP014169.1) were separated from the RAST output file and visualized using CIRCOS (Circular Genome Data Visualization).

### 4.4. Clusters of Orthologous Groups (COG) Analysis

A total of 159 *Sphingomonas* strains including *S. panacis* DCY99^T^ were annotated by the RAST (Rapid Annotation using Subsystem Technology) server. The 159 annotated amino acid sequence FASTA files were used for BPGA-1.3 (Bacterial Pan Genome Analysis) analysis. For orthologous clustering of functional genes, the USEARCH clustering algorithm. A 50% sequence identity was used as the cut-off value to generate a Core-Pan plot. For pan-genome functional analysis, COG and KEGG pathway distribution were used. The COG and KEGG IDs were assigned to all representative protein sequences from each orthologous protein cluster based on protein BLAST against reference COG and KEGG databases. Subsequently, the percent frequencies of COG and KEGG categories were calculated for core genes, accessory genes, and unique genes [100]. Then, pan-phylogenetic tree was visualized using dendroscope [114].

### 4.5. Genome Comparisons

USEARCH program was used for detailed comparative analysis of *S. panacis* DCY99^T^ with *S.* sp. LK11, *S. paucimobilis* *EPA505, P. savastanoi*, *E. cloacae*, and *A. brasilense*. *S.* sp. LK11 (CP013916.1) and *S. paucimobilis* EPA505 (NZ_BBJS00000000.1) genome were downloaded from NCBI database. Indole acetic acid pathway genes of *P. savastanoi* (P06617; Tryptophan 2-monooxygenase, P06618; Indoleacetamide hydrolase), *E. cloacae* (P23234; Indole-3-pyruvate decarboxylase) and *A. brasilense* (P51852; Indole-3-pyruvate decarboxylase) were downloaded from Uniport. Genes responsible for plant growth-promoting traits such as PAHs degradation and phenolic compound degradation were constructed to the USEARCH database. Comparative genome alignments were based on results of USEARCH algorithm with constructed database, identification of conserved gene order and construction of gene clusters.

### 4.6. In Vitro Plant Growth-Promotion and Product Assays

To assess indole acetic acid production, we followed the method as previously described [115], with some modifications for *in vitro* indole acetic acid production. At first, King B broth was used with and without additional L-tryptophan (3 g/L) [116]. After 6 days of incubation, indole acetic acid production was measured using a colorimetric method (Salkowski reagent). The concentration of indole acetic acid was also prepared to make the standard curve, the absorbance of bacterial supernatant was measured at 540nm for indole acetic acid production, the more different metabolites of indole acetic acid should be analyzed by LC with standards in further study. Qualitative testing of phosphate-solubilizing ability was checked by plate screening methods using a formulated medium [117]. A clear halo region around colonies in the opaque Pikovskaya medium indicated positive results for phosphate solubilization.

### 4.7. S. panacis DCY99^T^ and P. ginseng Meyer Compatibility under Biotic /Abiotic Stress

Surface-disinfected 2-year-old ginseng roots were used for ginseng pot assays. A ginseng pot assay was started by dipping the ginseng roots in *S. panacis* DCY99^T^ suspensions at various ODs (indicating variations in CFUs/mL) for 10 min, followed by cultivation in sterilized artificial soil consisting of vermiculite: perlite: peat moss at a 3:1:1 ratio with additional sterilized tap water [25% (*v/v*)] in pots (11 cm high and 11 cm diameter). We performed for the compatibility of strain DCY99^T^ in 2 years ginseng roots and analyzed priming effect against iron stress. Two-year-old *P. ginseng* roots were rinsed with tap water and twice rinsed with sterilized DW. Ginseng roots were treated with 500 mM FeCl_3_6H_2_O infected with *F. solani* and *I. mors-panacis* HB11 (KACC 44660) at 25 °C, respectively. We observed symptom development for 7 days after inoculation and then created a symptom severity scale. Each pot contained 5 roots. Each treatment was replicated in three pots.

### 4.8. GC-TOF-MS Analysis

Freeze-dried ginseng roots were prepared as powder. Then, 10 mg powder was extracted with 1 mL 80% methanol followed by homogenization using a mixer mill at 30 Hz/s for 10 min (Retsch MM400, Haan, Germany). The mixture was centrifuged at 13,000 rpm for 5 min, and the supernatant (100 μL) was vacuum dried using a speed vacuum concentrator (BioTron, Seoul, Korea). The extract was dissolved in 80% methanol (final concentration, 0.2 mg/mL), filtered using a 0.2-μm PTFE filter, and then measured by GC-TOF-MS (Supplementary Table S5). For each root, 10 biological and 3 analytical replicates were analyzed for organic acids including 3-hydroxybutanoic acid.

### 4.9. Constructing the Heat Map

The sphingolipid pathway genes of alpha-proteobacteria was downloaded from the KEGG and NCBI databases. Homologs for the serine palmitoyl transferase (EC:2.3.1.50) from *S. panacis* DCY99^T^ was found in the NCBI database. USEARCH was used to compare strain DCY99^T^ with the other 158 *Sphingomonas* strains and to select orthologs with the highest identity. As a result, Conserved genes were visualized using a heat map made with Python matplotlib.

### 4.10. Primer Design 

For the first screening, 300 bp of the *spt* gene were collected from NCBI and analyzed for primer possibilities. Two sets of primers were designed and evaluated for species-specific markers (Table 4). After optimization of the annealing temperature range from 50–65 °C, the best PCR conditions were as follows: initial denaturation at 95 °C for 3 min, 30 cycles of 95 °C for 30 s, 50–65 °C for 30 s, and 72 °C for 1 min followed by a final elongation at 72 °C for 5 min. PCR was conducted using 100 ng of *S. panacis* DCY99^T^ genomic DNA in a 25-μL total reaction volume of Genotech^®^ 2× Green PreMix (Genotech, Daejeon, South Korea). The PCR mix included 1 µL DNA template (100 ng/µL), 1 µL each primer pair, 10 µL Green PreMix, and 7 µL sterile DW for a 20 µL total reaction mix. We verified species-specific primers for *S. panacis* DCY99^T^ compared to other strains including DNA templates from *S. panaciterrae* DCY91^T^, *S. mucosissima*, *S. dokdonensis*, *S. xinjiangensis*, *S. faeni*, *S. aurantiaca*, and *S. aerolata.* Template DNA only and primer only PCR reactions were used for controls.

### 4.11. Primer Alignment

Nucleotide FASTA files of 159 *Sphingomonas* strains were aligned using ClustalW based on the *spt* primer product. The 10 *Sphingomonas* strains (*Sphingomonas* sp. Cra20, *Sphingomonas* sp. LM7, *Sphingomonas* sp. IBVSS2, *Sphingomonas* sp. ERG5, *Sphingomonas* sp. NAT212, *Sphingomonas* sp. UBA4815, *Sphingomonas hengshuiensis* WHSC-8, *Sphingomonas pruni* NBRC 15498, *Sphingomonas pruni* NBRC 15500, *Sphingomonas paucimobilis* LCT-SP1), which has notably different sequences, were used.

### 4.12. Quantification of S. panacis DCY99^T^ Gene Expression

We used cell suspensions with different OD values as templates (1 μL) in a 15-μL total reaction volume of SYBR^®^ Green SensiMix Plus Master Mix (Watford, England) and performed quantitative real-time PCR (qRT-PCR). Amplification, detection, and data analysis were carried out using a CFX 96/Connect Real-Time PCR system (Bio-Rad, Seoul, South Korea). We used the following thermal cycler conditions: 2 min at 95 °C followed by 40 cycles of 95 °C for 30 s, 64 °C for 50 s, and 72 °C for 1 min. The threshold cycle (Ct) was recorded, and the correlation between OD and Ct values is shown as an exponential regression.

## Figures and Tables

**Table 1:** Phosphate metabolism of *S. panacis* DCY99^T^.

Gene	Locus	Product
*phoB*	AWL63_12720	Phosphate regulon transcriptional regulatory protein PhoB
*phoR*	AWL63_12750	Phosphate regulon sensor protein PhoR
*phoU*	AWL63_12725	Phosphate transport system regulatory protein PhoU
*pstB*	AWL63_12730	Phosphate ABC transporter, ATP-binding protein PstB
*pstA*	AWL63_12735	Phosphate ABC transporter, permease protein PstA
*pstC*	AWL63_12740	Phosphate ABC transporter, permease protein PstC
*pstS*	AWL63_12745	Phosphate ABC transporter, substrate-binding protein PstS

**Table 2:** Metal tolerance of four *Sphingomonas* strains.

Metals	DCY99^T^	*S. oligophenolica*	*S. mali*	*S. pruni*
**No metals**	+++	+++	+++	+++
**Iron**	++	++	++	++
**Zinc**	++	++	++	w
**Cupper**	+	w	+	++
**Silver**	w	w	-	w
**Manganese**	++	++	++	++

**Table 3:** PAHs and 4-hydroxybenzene degradation pathway of *S. paucimobilis* EPA505.

Locus Tag	Catechol Meta Cleavage Pathway	Identity
BV96_03589	Catechol 2,3-dioxygenase	30%
BV96_02173	2-hydroxymuconic semialdehyde hydrolase	61%
BV96_03586	2-keto-4-pentenoate hydratase	50%
BV96_00245	4-hydroxy-2-oxovalerate aldolase	82%
Locus tag	4-hydroxybenzene Degradation Pathway	Identity
BV96_00588	P-hydroxybenzoate hydroxylase	84%
BV96_00594	Protocatechuate 4,5-dioxygenase alpha chain	80%
BV96_00593	Protocatechuate 4,5-dioxygenase beta chain	82%
BV96_00592	4-carboxy-2-hydroxymuconate-6-semialdehyde dehydrogenase	85%
BV96_00600	2-pyrone-4,6-dicarboxylic acid hydrolase	57%
BV96_00599	4-oxalomesaconate tautomerase	79%
BV96_00595	4-oxalomesaconate hydratase	82%
BV96_00598	4-carboxy-4-hydroxy-2-oxoadipate aldolase	84%

**Table 4:** Sequence information for probe and primers in this study.

Sequence Definition	Sequence Length	Probe Sequence	Genomic Position	Length	Tm	GC%
	1261	TCACATCGGCGTCGAACGTCATGCCCA	1006	27	78.8	59.3
Sequence Definition	Product Length	Sense/Anti-sense Primer	Genomic Position	Length	Tm	GC%
	149	CGTCTTCGCAGTCCTTGTG	907	19	67.4	57.9
		CCTGCTCGGCACCTACAA	1055	18	68.4	61.1

## Supplementary Materials

The following are available online at https://www.mdpi.com/1422-0067/21/6/2019/s1, Supplementary Figure S1. Scatter blot of genome size between number of coding sequence (CDS) from 159 *Sphingomonas* genomes. Supplementary Figure S2. Core-pan genome plot of the 159 *Sphingomonas* strains, Supplementary Figure S3. Phylogenetic tree of the 159 *Sphingomonas* strains, Supplementary Figure S4. D-gluconic acid biosynthesis and 3-hydroxybutanoic acid degradation pathway from *S. panacis* DCY99^T^, Supplementary Figure S5. Pot assessment of iron tolerance in *P. ginseng*, Supplementary Table S1. Detailed genome annotation of *S. panacis* DCY99^T^ using RAST, Supplementary Table S2. Detailed heavy metals related genes of four *Sphingomonas* strains, Supplementary Table S3. Result of BPGA from 159 *Sphingomonas* strains, Supplementary Table S4. Result of BPGA from 15 *Sphingomonas* strains, Supplementary Table S5. Result of GC-TOF-MS from healthy and rusty *P. ginseng*.
