# Supplementary material for: Comprehensive Genome Analysis on the Novel Species Sphingomonas panacis DCY99T Reveals Insights into Iron Tolerance of Ginseng
Source: Int J Mol Sci. 2020 Mar 16;21(6):2019. doi: 10.3390/ijms21062019 (PMC7139845; doi:10.3390/ijms21062019)
Supplement: Supplementary file 1 [file ijms-21-02019-s001.zip › Supplementary figure legends.docx]

**Supplementary figure legends**

**Supplementary Fig. S1.** Scatter blot of genome size between number of coding sequence (CDS) from 159 *Sphingomonas* genomes which were downloaded from NCBI database and estimated from RAST (Rapid Annotation using Subsystem Technology) server.

**Supplementary Fig. S2.** Core-pan genome plot of the 159 *Sphingomonas* strains.

**Supplementary Fig. S3.** The 159 strains of *Sphingomonas* were constructed into the phylogenetic tree due to Dendroscope. Each node name belongs to the name of the strain. It is clearly observed how strains gather into a cluster, which is more closely related than others. As for the strain DCY99, it falls into the closest relationship with the strain NRF15.

**Supplementary Fig. S4. (A)** D-gluconic acid biosynthesis pathway encoded by *S.* *panacis* DCY99^T^. **(B)** Results of GC-TOF-MS for 3-hydroxybutanoic acid from epithelium and roots of healthy ginseng and rusty-ginseng (Hp, Healthy ginseng epithelium; Hs, Healthy ginseng root; Rp, Rusty ginseng epithelium; Rs, Rusty ginseng root). **(C)** 3-hydroxybutanoic acid degradation pathway from *S. panacis* DCY99^T^.

**Supplementary Fig. S5. (A)** Pot assessment of iron tolerance in *P. ginseng*, morphological appearance of P. ginseng in response to Fe stress. Pot assay was observed by morphological alterations after 7 days, each pot included three seedlings. 1) Control; 2) iron stress at 250 mM; 3) iron stress at 500 mM; 4) iron stress at 1000 mM. **(B)** Biomass (fresh and dry weights) of P. ginseng seedlings under 500 mM of iron stress. FW, fresh weight; DW, dry weight.
